# Experimental Validation of a Micro-Extrusion Set-Up with In-Line Rheometry for the Production and Monitoring of Filaments for 3D-Printing

**DOI:** 10.3390/mi14081496

**Published:** 2023-07-26

**Authors:** João Sousa, Paulo F. Teixeira, Loïc Hilliou, José A. Covas

**Affiliations:** Institute for Polymers and Composites, University of Minho, 4800-058 Guimarães, Portugal; duarte.98@hotmail.com (J.S.); p.teixeira@dep.uminho.pt (P.F.T.)

**Keywords:** micro-extrusion, slit rheometer, 3D printing, cyclic olefin copolymer

## Abstract

The main objective of this work is to validate an in-line micro-slit rheometer and a micro-extrusion line, both designed for the in-line monitoring and production of filaments for 3D printing using small amounts of material. The micro-filament extrusion line is first presented and its operational window is assessed. The throughputs ranged between 0.045 kg/h and 0.15 kg/h with a maximum 3% error and with a melt temperature control within 1 °C under the processing conditions tested for an average residence time of about 3 min. The rheological micro slit is then presented and assessed using low-density polyethylene (LDPE) and cyclic olefin copolymer (COC). The excellent matching between the in-line micro-rheological data and the data measured with off-line rotational and capillary rheometers validate the in-line micro-slit rheometer. However, it is shown that the COC does not follow the Cox–Merz rule. The COC filaments produced with the micro-extrusion line were successfully used in the 3D printing of specimens for tensile testing. The quality of both filaments (less than 6% variation in diameter along the filament’s length) and printed specimens validated the whole micro-set-up, which was eventually used to deliver a rheological mapping of COC printability.

## 1. Introduction

Additive manufacturing (AM), also known as 3D printing, encompasses a vast and growing number of techniques whereby structures are built layer-by-layer without the use of molds [1,2]. AM can produce parts with significant geometrical complexity, either as prototypes for concept validation during the design stage, or as final products with advanced functionalities, for example, with gradient properties by criteriously combining several materials [3,4]. AM has been often considered a potentially disruptive technology, given its potential impact on the structure and operations of international business [5].

Among the available AM techniques for polymers, material extrusion is the most popular, being cheap, simple, compatible with the production of a small series, and offering good reproducibility. The fused deposition modeling (FDM) and fused filament fabrication (FFF) variants use previously extruded thermoplastic filaments that are melted and pushed through a nozzle to form thin threads that are subsequently deposited onto a print bed, and create successive horizontal layers (sections) of the part in the vertical direction.

Despite the growing practical utilization of FDM/FFF, the current range of commercially available materials in filament form remains relatively limited. Also, a minimum amount of filament (supplied in reels) must be purchased from manufacturers or distributors, even if only a single part with a small volume is to be manufactured.

In order to increase the number of suitable materials for FDM/FFF, it is of special interest to combine the properties of different polymers by blending them, reinforcing polymer matrices, or adding additives with specific functionalities. Twin-screw extruders, particularly of the co-rotating type, are commonly used for this purpose, as they possess excellent mixing capability, good yield, and have a modular construction, which allows for adopting the most appropriate screw and barrel configuration for each application. However, these machines have moderate pumping characteristics, which may affect the dimensional tolerance of the extrudates and may generate significant viscous dissipation [6]. In this context, counter-rotating twin-screw extruders offer good mixing and excellent pumping capacity, providing better control of the output, even when compared with the single-screw extruders usually found in commercial filament extrusion lines. Also, heating by conduction is predominant over viscous dissipation, which makes these machines especially adequate for processing thermally sensitive materials, such as poly(vinyl chloride) (PVC) [7,8]. Again, high precision in output directly translates into the extrusion of filaments with well-defined dimensions.

Small-scale equipment is ideal to cope with the small amounts of research-grade materials available for development purposes. Micro-extrusion must follow the main principles and functionalities of equivalent industrial lines in terms of the thermomechanical environments created and associated residence times, although at a much smaller scale [9]. Taking a step further, the determination of the shear viscosity at relatively high shear rates, that is, in the region of practical extrusion processing (greater than 10 s^−1^), can become accessible by coupling an instrumented die to the micro-extruder. This is of great practical interest for the characterization of polymer melts and the prediction of their processability, and provides the link between polymer properties, processing parameters, and final product characteristics [10]. In the specific case of FDM/FFF, coupling an in-line rheometer to a filament extrusion line contributes to the rheological understanding of this printing process and adds to the toolbox of approaches that include instrumenting printers [11,12,13] aiming to better map the materials’ printability–rheological properties relationships [14].

Therefore, in order to promote the research, development, and production of new materials for FDM using small material samples, and also contribute to establishing correlations between process parameters, materials rheology, and the printability of an extruded filament, a micro-filament extrusion line was designed and built. The equipment consisted of a micro-counter-rotating twin-screw extruder, an in-line slit rheometer, a filament die, miniaturized material feed, and downstream equipment. The in-line slit micro-rheometer was designed for minimizing the time delay between the decision to make a measurement and obtaining the result and, equally important, for avoiding the need to subject the material to further thermal/flow cycles associated with the preparation of samples for conventional laboratory rheological measurements, which may affect its initial characteristics. Moreover, it takes on board the results from a previous study that demonstrated that small piezoelectric sensors are effective in accurately measuring steady melt pressures during polymer extrusion [15]. The rheological data obtained in-line and off-line were directly confronted. The operating window of the micro-filament extrusion line was established, and its capacity to produce suitable filaments for FDM/FFF was demonstrated by assessing the properties of 3D-printed specimens.

## 2. Materials and Methods

Two materials were selected based on their melt processability at temperatures lower than 200 °C (see below). An extrusion-grade LDPE (ALCUDIA^®^ LDPE 2221FG, from Repsol, Spain) with a density of 0.922 g/cm^3^, a melt flow index of 2.1 g/10 min (190 °C/2.16 kg), and a processing temperature range between 150–180 °C was used for the assessment of the operational window of the micro-extrusion line and for the first tests for the validation of the micro-rheometer. A COC (Topas^®^ COC 8007S, from Topas Advanced Polymers, Oberhausen, Germany) with a density of 1.010 g/cm^3^, a melt flow index of 1.7 g/10 min (190 °C/2.16 kg) [16], and a processing temperature range between 190 and 250 °C was used for the validation of the micro-rheometer, for the production of filaments, and for 3D printing.

A stress-controlled rotational rheometer (ARG2, TA Instruments, New Castle, DE, USA) was used with 25 mm parallel plates and disks compression-molded from LDPE and COC (10 tons for 2 min at 180 °C and 190 °C, respectively). All tests were carried out at 180 °C and 190 °C for LDPE and COC, respectively. Oscillatory time sweep tests were first performed at 1 Hz and a strain of 0.1% to assess the materials’ thermal stability. Then, fresh disks were loaded and frequency sweeps were performed to record the mechanical spectra of the materials using oscillatory strains of 0.01% and 0.1%. The superimposition of the spectra measured at these two different strains ensured that tests were performed in the linear viscoelastic regime. Finally, new disks were loaded to measure the flow curves of the materials by sweeping the shear rates from 0.01 s^−1^ to 10 s^−1^ and considering a shear viscosity as steady when readings were less than 5% over 10 s. The duration of each test was less than 20 min, which ensured the thermal stability of the materials. Additional testing was performed with COC to infer possible slip effects at the COC/shearing plate interface. Different gaps were tested with disposable grooved aluminum plates (with a diameter of 40 mm).

Flow curves were also measured with capillary rheometry (Davenport rheometer from Daventest Ltd., London, UK) to access the shear rates of interest for in-line micro-slit rheometry, filament extrusion, and FDM/FFF. The rheometer was coupled to a Dynisco PT422A melt pressure transducer connected to a Dynisco 1390 strain gauge indicator interfaced to a data-acquisition system NI-9215 from National Instruments and driven by a custom-written LabVIEW routine. For each material, two flow curves were measured with two dies having a radius of 1 mm, but different lengths (10 and 20 mm), in order to perform the Bagley correction for the measured pressure drop and thus remove the additional pressure drop effects associated with the edge effects (die entrance and exit). In addition, the Weissenberg–Rabinowitsch correction for the apparent shear rate was implemented to take into account the non-Newtonian nature of the polymer melts.

For the validation of the COC filaments manufactured by the micro-extrusion line, tensile specimens (50 mm length and 2 mm thickness, according to the DIN 53504 standard) were printed with a Raise3D Serie Pro2 printer (Raise3D, Irvine, CA, USA) equipped with a 0.4 mm nozzle. In this particular case, the model required a total of 10 layers to be printed. The planning and slicing of the latter were executed using the ideaMaker software developed by Raise3D. Specifically, the software was used to configure the print settings, generate the required toolpaths, and optimize the overall print quality. The layer orientation was set at 45 degrees and the layer height was 0.2 mm, ensuring a fine level of detail in the finished object, as well as an infill degree of 100%. A universal testing machine INSTRON 4505 was used for the mechanical testing of the printed test specimens.

## 3. Filament Micro-Extrusion Line and In-Line Micro-Slit Rheometer

**Figure 1:**
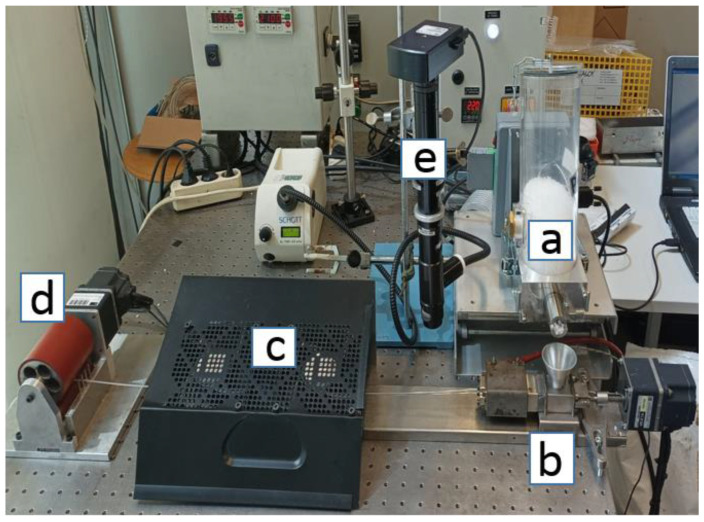
The micro-extrusion line used to produce filaments for FDM: mini-volumetric feeder (**a**) micro-extruder fit with a capillary die (**b**), cooling fans to solidify the filament (**c**), which is stretched by a haul-off (**d**). An optical microscope (**e**) is inserted in the line for the visualization of the extruded hot filament.

Figure 1 presents the layout of the filament micro-extrusion line. Pellets (typically, spheres or rounded cubes with sizes of approximately 3 mm) of the material to be extruded were fed to the hopper of the micro-extruder by a mini-volumetric feeder (based on a Piovan MDP1 feeder, Italy, fitted with a miniaturized screw and barrel) (a) at a pre-defined rate, thus setting the throughput of the process. This was of the order of 50–150 g/h, which is one order of magnitude lower than that of laboratory extruders and 1000–5000 times smaller than that of industrial machines. The intermeshing counter-rotating twin-screw micro-extruder (b) dragged forward axially, and melted and pumped the material through a circular die, so that a filament was continuously generated. As it exited the die, the filament was cooled down by an air-blowing unit consisting of two fans (c), while being taken up by a haul-off (d) that defined the line speed and allowed for controlling the filament diameter. A digital polarized optical microscope (e) (a long-distance 20× objective—ELWD 20X/0.42, Edmund Optics, York, England—mounted on a lens tube—210200 InfiniTube Standard, Proximity series, Infinity, Göttingen, Germany—attached to a highspeed monochrome USB 2.0 camera—LU165M-IO, Lumenera, Ottawa, ON, Canada) was added to the line to assess the physical quality of the filament and the residence times.

**Figure 2:**
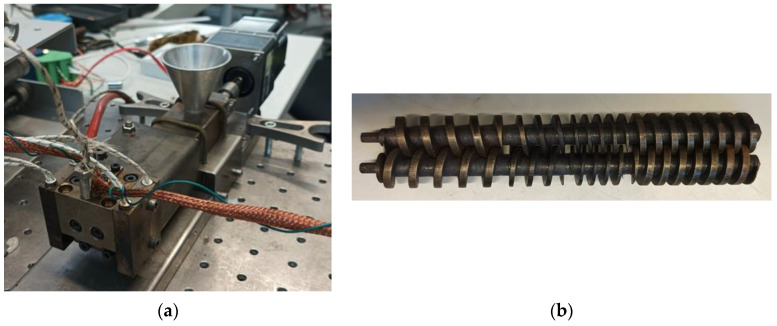
The intermeshing counter-rotating micro-twin-screw extruder: (**a**) general view of the die, barrel, and drive unit; (**b**) counter-rotating parallel screws.

The fully intermeshing counter-rotating micro-twin-screw extruder (coupled to the die) is illustrated in Figure 2. The screws were driven by a brushless DC motor (BLFM230-GFS, OrientalMotor, Düsseldorf, Germany) via a gear unit, which transmitted the movement of the motor shaft to the two screws. The maximum velocity was 20 rpm, which was typical for this type of machine and resulted from the need to minimize the forces generated at the intermeshing zone between screws, which pushes them against the barrel wall, thus causing premature wear. The gear unit contained helical gears, as they provided more precise and nearly noiseless rotation, and could withstand higher loads when compared with straight gears. The barrel was made of steel and contained a ceramic thermal barrier near to the feed opening and hopper in order to reduce the heat transferred from downstream, which could cause the sticking of the material in the feed throat and thus hamper the entry of pellets to the screw channels. The barrel was heated by a rectangular heating resistance and controlled by an Omron E5CB Controller using a type J thermocouple for monitoring purposes. Different extrusion dies could be directly coupled to the front of the barrel. The counter-rotating screws (Figure 2b) were 120 mm long, with an external diameter of 14 mm, and had four distinct geometrical zones with different pitches and flight thicknesses, in order to drag the solids forward, progressively heat and melt them, and finally generate pressure for pumping. If the effect of the unavoidable mechanical gaps between the screws and the screws and the barrel was discarded, the extruder worked as a positive displacement pump, with the material moving axially inside individual C-shaped channels. Moreover, the extruder was starve-fed; hence, at a constant throughput defined by the feeder, the number of fully-filled screw channels varied with screw speed.

**Figure 3:**
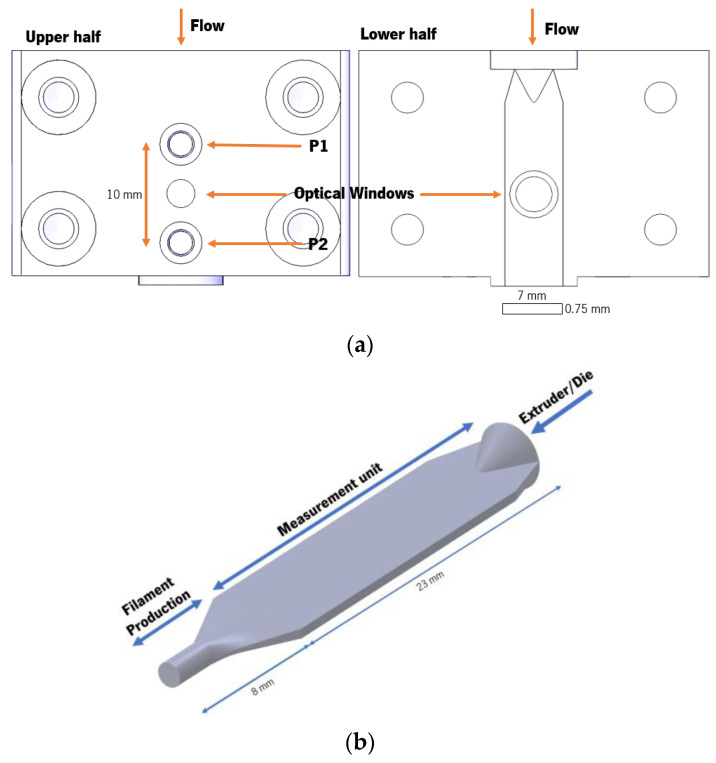
The micro-slit rheometer: (**a**) the two modules (rheological slit and shaping die; (**b**) schematic showing the location of the piezoelectric transducers and optical windows in the slit.

The micro-slit rheometer is schematized in Figure 3. The device comprised two modules (Figure 3a), the rheological slit and a shaping die to convert the 23 × 7 × 0.75 mm slit into a filament with 1.75 mm diameter (the standard dimension used by most commercial 3D printers). The measuring slit module contained two holes separated by 10 mm to accommodate the piezoelectric sensors. These had a diameter of 2.5 mm (i.e., much smaller than that of the usual melt pressure transducers), a pressure range from 0 to 20 MPa, and a temperature range from 0 to 200 °C. Although they generated a signal drift with time, their suitability for in-line slit rheometry was previously demonstrated [15]. The rheological slit was also equipped with two directly opposite optical windows to allow for the rheo-optical characterization of essentially non-turbid materials, such as polymer blends [10] and nanocomposites [17]. The temperature control of the micro-slit rheometer was independent of the extruder and was achieved by four heating cartridges. The temperature was read by a type J thermocouple and controlled by an Omron E5CB temperature controller.

## 4. Results and Discussion

### 4.1. Operational Window of the Set-Up

The throughput stability, temperature control, and residence time in the micro-extruder were studied by varying a set of processing parameters in the series of tests summarized in Table 1 and carried out with the LDPE. The minimum feed rate available with sufficient precision was 45 g/h for the feeder and LDPE pellets (for material in powder form, a lower feed rate would be possible). This minimum feed rate allowed for a smooth extrusion of filament with a screw speed of 5 rpm, thus defining the lower limit of the operational window of the set-up. The throughput stability was assessed by weighting extrudates for 15 min for each condition, which allowed for the computation of an average mass. To compare the melt temperature *T_melt_* with the set temperature *T_set_* of the barrel, a temperature probe was placed in the die each minute during the 15 min of testing. An average $\Delta T=\left|T_{melt}-T_{set}\right|$ was thus computed. The residence time was determined by introducing a masterbatch tracer at the feed hopper and measuring the time required for it to reach the die exit (minimum residence time) and the time required for the masterbatch to disappear from the extrudate (maximum residence time) from the analyses of the polarized optical microscope images taken on-line.

The measured throughputs *Q_T_* and Δ*T* during all tests are reported in Table 1. As expected, the feeder and extruder throughputs were identical, with 3% average error, the latter being associated with the precision of the feeder. Also, the extrusion line was capable of maintaining a rather constant throughput, regardless of the operating conditions, as average errors of approximately 2% were observed. The temperature difference between the melt temperature and set temperature was always under 1 °C for all tested processing conditions (the largest Δ*T* was measured when the set temperature was lower, at 170 °C, in Table 1). This not only validated the temperature-control capability of the extruder, but also demonstrated that viscous heating was nearly insignificant, which is a well-known advantage of these types of extruders, especially for processing thermally sensitive materials (which is often the case for biodegradable polymers).

**Figure 4:**
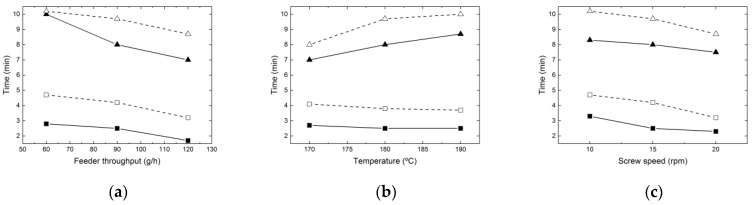
Minimum and maximum residence times in the micro-extruder and die measured when varying: (**a**) the feeder throughput; (**b**) the barrel temperature; (**c**) the screw speed. Solid symbols are the minimum times, whereas empty symbols are the maximum times measured without (squares) and with (triangles) the rheological die.

The minimum and maximum residence times measured in the micro-extruder and die are shown in Figure 4, together with the times measured when the micro-slit rheometer was coupled to the extruder. It is worth noting that, due to the usual variation in the residence time distribution in screw extruders, the average residence time was close to the minimum value, which varied between 2 and 3 min, depending on the operating conditions, whilst the maximum residence time reached 11 min. Adding the rheological die augmented the residence time by approximately 90 s. As expected, as the feeder throughput or the screw speed were increased, the residence time decreased. In the first case, the number of fully filled channels increased and the melt pressure started increasing earlier in the screw and reaching higher values at the screw tips, hence forcing larger flows through the die. When the screw speed was increased at a constant output, the number of fully filled channels actually decreased, but the material advanced faster along the screw axis. The effect of the barrel temperature on the distribution of the residence times was more complex to predict, as it was influenced by the efficiency of melting, as well as by the viscosity of the resulting melt, which may affect the local flow patterns. Overall, the distribution was broader when the temperature was increased, as the longest residence time was enhanced, whereas the minimum was virtually unaffected. For example, the reduced melt viscosity facilitated backflow through the mechanical gaps, inducing a longer time in the extruder, but also a higher level of distributive mixing.

### 4.2. Validation of the In-Line Micro-Slit Rheometer

A full treatment of the experimental protocol and data treatment for in-line slit rheometry has been provided elsewhere [18]. Briefly, the principle of slit rheometry is based on the pressure drop associated with the flow of material in the slit, which is proportional to the shear stress and is directly measured by the two piezoelectric sensors, whereas the material shear rate is controlled either by the screw speed or the feeder throughput. The use of piezoelectric sensors enables the miniaturization of the slit, but adds complexity associated with the electronic drift of the voltage, which is inherent to the piezoelectric effect. Thus, the acquired signals need to be appropriately corrected before incorporating the corresponding pressure measurements into rheological equations. The protocol for the piezo electrical drift correction to the pressure measurements has also been detailed elsewhere [15].

**Figure 5:**
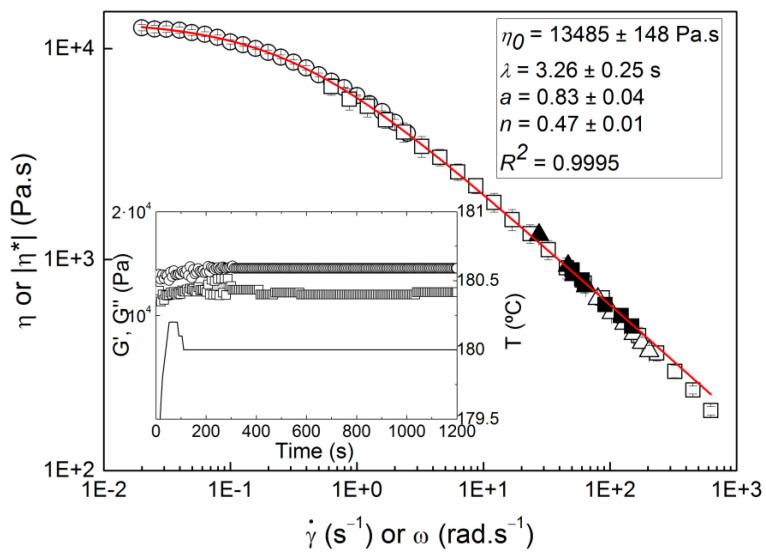
LDPE flow curves measured at 180 °C with the micro-in-line slit rheometer (solid symbols) and varying the feeding throughput (squares) or the screw speed (triangles), with the rotational rheometer in steady shear (open circles) or in oscillatory shear (open squares), and with the capillary rheometer (open triangles). The line is the fit of equation 1 to the whole set of data with the adjusted parameters displayed in the legend. Inset: thermal stability of LDPE at 180 °C inferred from a time sweep performed in the rotational rheometer: G′ (squares), G″ (circles), and temperature T (line) measured as a function of time.

The flow curve measured with LDPE using the micro-in-line slit rheometer is presented in Figure 5, together with the flow curves obtained with off-line rotational and capillary rheometries. Overall, all data showed a quite satisfactory overlap, which indicated that the micro-rheometer performed well. The experimental errors for each data point in the flow curve varied between 3.1% and 5.0% when varying the screw speed, against 1.4% and 4.5% when varying the feeder throughput. In addition, even the highest possible residence time (11 min) of LDPE in the micro-extruder had no impact on the data, which was to be expected, as LDPE is thermally stable for at least 20 min (see inset in Figure 5). As such, the LDPE flow curve measured in-line did not depend on the extrusion conditions, namely changing the feeding throughput or the screw speed to vary the shear rates in the measuring slit. This is in contrast to more rheologically complex materials, such as polymer blends or nanocomposites, as well as thermally unstable polymers, which may require a specific double-slit rheological die to by-pass the effects of different thermo-mechanical histories on the measured flow curves [19]. The quality of the rheological measurements was mirrored in the quality of the fit (quantified by a coefficient of determination *R*^2^ for the least-squares method of 0.9995) of the Carreau–Yasuda equation to the experimental flow curves and the mechanical spectrum of LDPE plotted in Figure 5. The equation reads as:

(1) $$\eta =\eta_{0}{\left(1+{\left(\lambda \dot{\gamma }\right)}^{a}\right)}^{\left(n-1\right)/a},$$

where *η*_0_ is the zero shear viscosity, *λ* is the characteristic relaxation time in a distribution of times with breath *a*, and *n* is the power law index describing the shear thinning behavior of the polymer melt. This equation is commonly used to model the flow of polymer melts [20]. The fact that the mechanical spectrum overlays the flow curve indicates that the Cox–Merz rule is obeyed for LDPE, which is expected for such a rheologically simple material [21].

**Figure 6:**
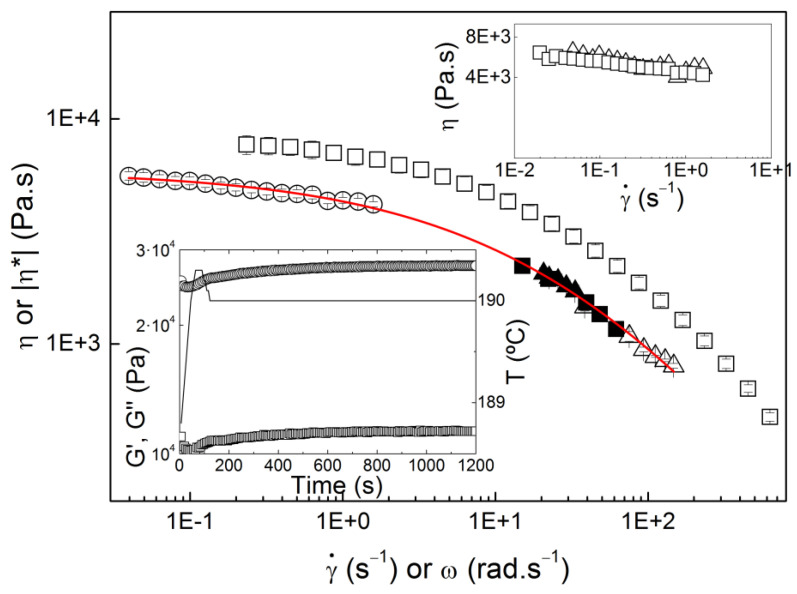
COC flow curves measured at 190 °C with the micro-in-line slit rheometer (solid symbols) and varying the feeding throughput (squares) or the screw speed (triangles), with the rotational rheometer in steady shear (open circles) or in oscillatory shear (open squares), and with the capillary rheometer (open triangles). The line is the fit of equation 1 to the whole set of data, with the following computed parameters: *η*_0_ = 5886 ± 123 Pa.s, *λ* = 0.019 ± 0.003 s, *a* = 0.46 ± 0.05 and *n* = 0.00 ± 0.007. Upper inset: comparison of steady flow curves measured with a 25 mm smooth plate (squares) and a 40 mm grooved plate (triangles). Lower inset: thermal stability of COC at 190 °C inferred from a time sweep performed in the rotational rheometer.

The validation of the micro-in-line slit rheometer was also performed with a more rheologically complex material. The various flow curves obtained for COC at 190 °C are compared in Figure 6. Both the trends of the curves and the good overlap of two data points acquired with the capillary rheometer with the data points measured with the micro-rheometer are noted, which suggests again that the processing conditions had no impact on the rheological data. This was, again, explained by the thermal stability of COC at 190 °C during 20 min (see lower inset in Figure 6, which reports the time-dependence of the shear dynamic moduli G′ and G″). As the flow curve measured in steady rotational shear did not overlap the flow curves measured with the micro-slit or capillary rheometer, the mechanical spectrum of COC is also plotted in Figure 6.

The mechanical spectrum lies above the flow curve, thus suggesting that the superposition of linear shear dynamic rheology and steady shear rheology was impossible for COC in the range of shear rate and frequency tested. In short, COC did not follow the Cox–Merz rule. Instabilities due to secondary flow or slip of material on the shearing walls are often responsible for deviations from the Cox–Merz rule [21]. Nevertheless, the absence of edge fracture during steady shear, as well as the good reproducibility of data measured with distinct shearing geometries (in both surface quality and size, see upper inset in Figure 6), ruled out any slip or instability. Thus, the lack of superimposition between the oscillatory and steady shear data suggested that COC did not obey the Cox–Merz rule. The COC chain rigidity inherent to the cyclic olefins in the copolymer probably explained the flow-induced orientation of the melt under steady shear, even at slow rates, resulting in a reduced shear viscosity [20]. Indeed, earlier rheological tests of COC carried out in micro-capillary dies showed a rheological complexity for this polymer melt [22], though pressure effects on the measured viscosity were clearly overlooked [23].

In order to bypass the lack of obeyance to the Cox–Merz rule, the analysis of the fit quality of equation 1 to the steady shear data was used to evaluate whether the rheological data measured with the micro-rheometer were solid. The fit is presented in Figure 6 and returns *R*^2^ = 0.9981, which compares well with the value achieved for LDPE, and thus validates the micro-rheometer, even for a rheologically complex material, such as COC.

### 4.3. Printability of the Extruded Filaments

COC filament production for 3D printing was carried out with the barrel and die set to 190 °C. The operating conditions were tuned to yield a filament with the required constant diameter, low ovality, and smooth surface. For example, the haul-off speed to draw down the filament must compensate for the extrudate swell at the die exit; otherwise, too-thick filaments would be produced. The optimized parameters used with the micro-extrusion line were a screw speed of 15 rpm, a feed rate of 120 g/h, and a haul-off speed of 750 mm/min. This resulted in a shear rate of 6.4 s^−1^ in the 4 mm capillary die and a draw-down ratio of 4.8. The filament produced had a diameter of 1.77 ± 0.05 mm (mean and standard deviation computed from 80 measurements along 16 m), good roundness, and a smooth surface. The standard deviation was within the range of commercially available filament reels.

**Figure 7:**
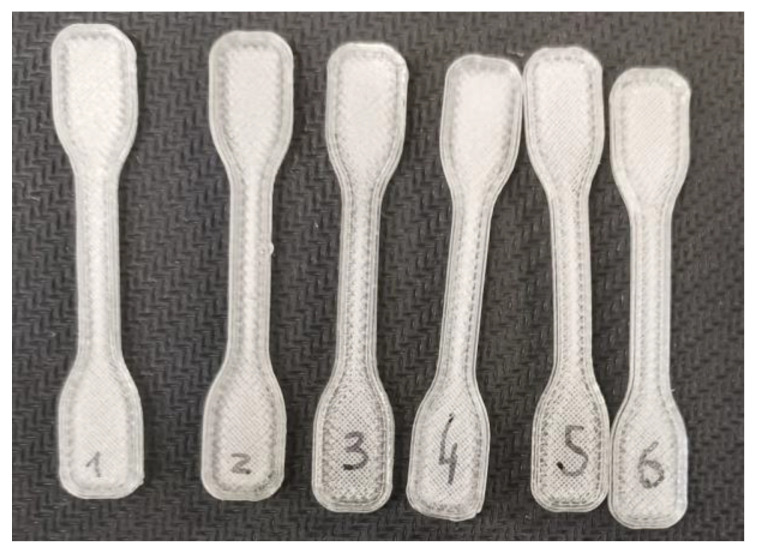
Tensile test specimens printed at 240 °C with a printing speed of 40 mm/s.

Table 2 presents the printing variables tested in order to obtain good-quality printed tensile test specimens. The 3D printer pulled the filament adequately, without any difficulties, such as slipping or abrasion, being observed. Nozzle temperatures ranging from 190 °C to 240 °C were tested. The lowest corresponded to the temperature used for the in-line rheological characterization of COC. However, at this temperature, printing speeds higher than 1 mm/s hindered the proper deposition of a COC thread on the build platform of the printer due to excessive viscosity. Higher printing temperatures facilitated the flow of COC melt in the nozzle, but only at 240 °C was it possible to attain good print quality, without voids. So, at this temperature, the printing speed was finally maximized. Figure 7 depicts the printed tensile test specimens with the required dimensional precision.

Table 3 presents the tensile properties of the 3D-printed COC specimens and compares them with those reported by the polymer manufacturer for injection-molded parts [24]. A relative difference can thus be computed for each mechanical property. As expected, injection-molded specimens performed better than the 3D-printed equivalents, due to the higher levels of molecular orientation of the former and the inherent porosity and poorer surface quality of the latter. The magnitude of the differences was well within those reported for other polymers, such as polypropylene [25], poly(lactic acid) [26], or polyethylene ketone ketone [27], thus validating the filaments for FDM/FFF.

### 4.4. Rheology-Printability Relationships

**Figure 8:**
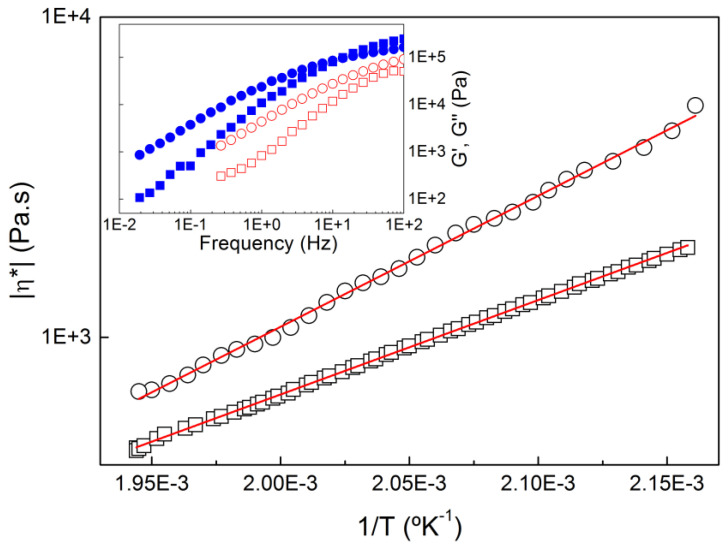
Temperature dependence of the dynamic viscosity |η*| of COC measured at 1 Hz (circles) and 10 Hz (squares). Lines are Arrhenius fits to the data. The inset shows the mechanical spectra (G′ squares, G″ circles) of COC measured at 190 °C (filled symbols) and 240 °C (open symbols).

With a view to relate the rheological properties of COC to its printability, additional off-line rheological characterization was performed at temperatures between 190 °C and 240 °C. In particular, the steady shear viscosity at shear rates $\dot{\gamma }$ between 5 s^−1^ and 262 s^−1^ was scrutinized, as this was the range of the shear rates achieved in the printer’s nozzle and between the nozzle tip and the platform for all tested conditions (see Table 2). In addition, other linear rheological parameters, such as the zero shear (Newtonian) viscosity *η*_0_ and the longest relaxation time of the melt *τ_R_*, were relevant for FDM/FFF [24,26,27,28]. Figure 8 presents the temperature dependence of the dynamic viscosity |η*| measured at a strain of 0.5% and for two frequencies tested in two separate temperature sweeps at 5 °C/min.

**Figure 9:**
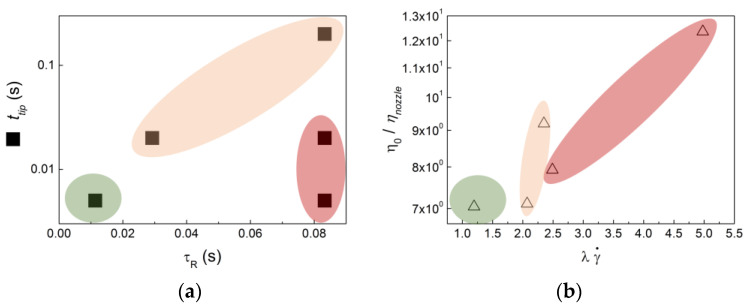
Rheological maps of the printability of COC for all printed conditions tested (see Table 2): (**a**) characteristic time of the flow under the nozzle tip *t_tip_* versus the longest relaxation time *τ_R_* of the deposited molten road; (**b**) normalized viscosity of the melt in the nozzle η_0_/*η_nozzle_* versus the Weissenberg number in the nozzle *λ*$\dot{\gamma }$.

As expected for the range of temperatures tested that were well above the glass transition, |η*| showed an Arrhenius dependence with the temperature. However, the barrier energy *E_a_* depended on the frequency as Arrhenius fits to the data returned *E_a_*_10_ = 56.4 ± 0.1 kJ/molK for 10 Hz against *E_a_*_1_ = 78.2 ± 0.7 kJ/molK for 1 Hz. This suggests that the COC Rouse dynamics at higher frequencies showed a different temperature dependence compared with the slowest reptation dynamics *τ_R_* at lower frequencies. This was more evident in the different mechanical spectra plotted in the inset in Figure 8, which could not be superimposed. This complex temperature dependence could also explain the rheological complexity mirrored in the fact that COC did not obey the Cox–Merz rule. Nonetheless, *E*_*a*10_ could be used to extrapolate the viscosities at play in both nozzle *η_nozzle_* and deposited bead *η_tip_* from the steady shear viscosities computed with equation 1 at the corresponding shear rates at 190 °C (see Figure 6), as fast segmental dynamics should essentially be at play under such a fast flow. *τ_R_* was estimated from the cross-over frequency between G′ and G″ [26], whereas η_0_ was taken from the terminal Newtonian regime in the mechanical spectra (see the plateau in |η*| at lower frequencies in Figure 6). Then, *E_a_*_1_ could be used to extrapolate both η_0_ and *τ_R_* at the corresponding printing temperature, as it was connected to slower (equilibrium) dynamics. Such extrapolations allowed for the construction of the rheological maps of COC printability proposed in Figure 9, where the linear viscoelastic characteristics of COC (η_0_ and *τ_R_*) were compared with the COC nonlinear viscoelastic properties in the nozzle *η_nozzle_* and with the characteristic processing times *t_tip_* and 1/$\dot{\gamma }$ for all printing conditions tested.

The maps presented in Figure 9 identify three regions. COC printability was satisfactory (green areas) when the COC melt showed fast reptation dynamics and lower viscosity ratio η_0_/*η_nozzle_*, which allowed for fast printing (the inverse of the shear rate under the nozzle tip gave a faster *t_tip_*), while the Weissenberg number in the nozzle *λ*$\dot{\gamma }$ was lower. Recall here that *λ* was the relaxation time of the COC melt computed from the fit of equation 1 to the COC flow curve (see Figure 6). In contrast with this, too-large Weissenberg numbers in the nozzle and a strong shear thinning melt (large ratio η_0_/*η_nozzle_*) were detrimental to any road deposition (red areas). Finally, a third area corresponding to a successful road deposition, but producing a specimen with voids (orange area), was identified at intermediate Weissenberg numbers and slower printing velocities. Note that such conditions also related to reptation dynamics *τ_R_* that nearly matched the characteristic printing time *t_tip_*. Thus, there was little or no polymer relaxation during deposition, as *τ_R_* was closer to *t_tip_* [29]. In addition, the polymer chains’ orientation and disentanglement in the nozzle were not significant, as *λ*$\dot{\gamma }$ was only slightly larger than 1.

However, the maps in Figure 9 do not inform about the significant drawing of the extruded melt upon deposition. Indeed, while one end of the thread emerged vertically downward from the nozzle, the other end was already deposited onto the platform or onto a previously deposited layer, which moved away horizontally at a high speed. Following the criteria set by McIlroy and Olmsted [29], the conditions for successful printing found in the present study corresponded to a significant melt elongation, as the printing speed was much larger than the linear speed of the extrudate. On the other hand, specimens with voids were printed with slower velocities, where no drawing should occur. Drawing, in turn, is known to affect the polymer chain relaxation and orientation [14], but such extensional effects were not captured in the maps displayed in Figure 9.

## 5. Conclusions

A micro-extrusion line consisting of a micro-counter-rotating twin-screw extruder, an in-line slit rheometer, a filament die, miniaturized material feed, and downstream equipment was designed, built, and experimentally validated. In-line rheological data measured for LDPE and COC were virtually superimposed with data obtained off-line with conventional methods, thus validating the methodology. The set-up was successfully used to produce COC filaments for subsequent 3D printing. FDM/FFF printing of standard tensile test specimens was also performed effectively. As expected, the mechanical performance of the printed specimens was lower than that of those obtained by injection molding due to their lower molecular orientation and inherent porosity. Finally, owing to the complex rheological behavior shown by COC, printability maps were established.

## Figures and Tables

**Table 1:** Tests performed to assess the operational window of the micro-extruder by varying the barrel set temperature *T_set_*, the screw speed *N*, or the feeder throughput *Q*. The temperature difference Δ*T* and the throughput *Q_T_* measured for each test are reported.

Test	Changed Parameter	*T_set_* (°C)	*N* (rpm)	*Q* (g/h)	Δ*T* (°C)	*Q_T_* (g/h)
0	Reference conditions	180	15	90	0.6 ± 0.1	93.0 ± 1.8
1	Temperature	170	15	90	1.0 ± 0.1	90.6 ± 1.2
2	Temperature	190	15	90	0.4 ± 0.1	93.0 ± 0.6
3	Screw speed	180	10	90	0.5 ± 0.1	89.4 ± 0.6
4	Screw speed	180	20	90	0.6 ± 0.1	93.6 ± 1.8
5	Feeder throughput	180	15	60	0.4 ± 0.2	62.4 ± 0. 6
6	Feeder throughput	180	15	120	0.6 ± 0.2	120.6 ± 2.4

**Table 2:** Printing tests performed to determine the set of printing parameters producing good-quality printed samples. Printing quality was quantified by the largest standard deviation in specimens’ dimensions computed from 6 samples. Tests resulting in no printed road on the platform are listed as *n.p.*, whereas printed specimens with large voids (*L.V.*) and small voids (*S.V.*) are also listed.

Printing Parameters	Test 1	Test 2	Test 3	Test 4	Test 5
Nozzle temperature (°C)	190	190	190	215	240
Printing speed (mm/s)	40	10	1	10	40
Shear rate in nozzle $\dot{\gamma }$ (s^−1^)	262	131	109	262	262
Shear rate in road	200	50	5	50	200
Printing quality (%)	*n.p.*	*n.p.*	*L.V.*	*S.V.*	7.5

**Table 3:** Tensile properties of the printed specimen at 240 °C and of the injected COC specimen.

	Printed	Injection-Molded ^1^	Difference (%)
Strength at yield (MPa)	51.45 ± 3. 67	63	18.3
Strain at yield (%)	3.64 ± 0.45	4.5	10.1
Young modulus (GPa)	1.82 ± 0.09	2.6	30

^1^ Retrieved from the COC table of specifications for injection-molded parts [19].

## Data Availability

The data presented in this study are available in the Masters Thesis of J.S. which can be shared on request from the corresponding author.
